# Co-Application of Silicate and Low-Arsenic-Accumulating Rice Cultivars Efficiently Reduces Human Exposure to Arsenic—A Case Study from West Bengal, India

**DOI:** 10.3390/toxics11010064

**Published:** 2023-01-09

**Authors:** Arkaprava Roy, Siba Prasad Datta, Mandira Barman, Debasis Golui, Somnath Bhattacharyya, Mahesh Chand Meena, Viswanathan Chinnusamy, Suchitra Pushkar, Punyavrat S. Pandey, Mohammad Mahmudur Rahman

**Affiliations:** 1 Division of Soil Science and Agricultural Chemistry, ICAR-Indian Agricultural Research Institute, New Delhi 110012, India; 2 Department of Civil, Construction and Environmental Engineering, North Dakota State University, Fargo, ND 58102, USA; 3 Bidhan Chandra Krishi Viswavidyalaya, Mohanpur, Nadia, West Bengal 741252, India; 4 Division of Plant Physiology, ICAR-Indian Agricultural Research Institute, New Delhi 110012, India; 5 Education Division, Indian Council of Agricultural Research, New Delhi 110012, India; 6 Global Centre for Environmental Remediation (GCER), College of Engineering, Science and Environment, The University of Newcastle, Callaghan, NSW 2308, Australia; 7 Department of General Educational Development, Faculty of Science & Information Technology, Daffodil International University, Ashulia, Savar, Dhaka 1207, Bangladesh

**Keywords:** arsenic, hazard quotient, rice cultivar, root-secreted organic acids, silicate

## Abstract

We investigated the effect of practically realizable doses of silicate on arsenic (As) uptake by differential-As-accumulating rice cultivars grown on geogenically As-polluted soil. The possible health risk from the dietary ingestion of As through rice was also assessed. In addition, a solution culture experiment was conducted to examine the role of root-secreted weak acids in differential As acquisition by rice cultivars. When grown without silicate, Badshabhog accumulated a much smaller amount of As in grain (0.11 mg kg^−1^) when compared to the other three varieties. Satabdi, IR-36, and Khitish accumulated As in grain beyond the permissible limit (0.2 mg kg^−1^) for human consumption. The application of silicate effectively reduced the As content in the grain, husk, and straw of all of the cultivars. The grain As content fell to 17.2 and 27.6% with the addition of sodium metasilicate at the rates of 250 and 500 mg kg^−1^, respectively. In the case of Khitish, the grain As content was brought down within permissible limits by the applied silicate (500 mg kg^−1^). The integrated use of low-As-accumulating cultivars and silicate has great potential to reduce the public health risks associated with As. A positive correlation between root-secreted total weak acid and grain As content could explain the different rice cultivars’ differential As acquisition capacity.

## 1. Introduction

Arsenic (As) contamination in the groundwater of the Bengal Delta Plain (BDP) has been widely recognized as the worst case of mass poisoning in human history, and it has resulted in 35–77 million people being exposed to risk on a land covering 0.173 million km^2^ [1]. Soils from some places of this region reportedly accumulate very high levels of total As (up to 52 mg kg^−1^) as compared to the global average of 5 mg kg^−1^ [2]. There are areas in BDP where 24.1% of the total death toll is attributed to As poisoning with an estimated annual fatality up to 43,000 [3]. Although As-laden drinking water has received most of the attention to date, As-tainted rice has emerged as an important conduit for passing the toxicant to mankind in the As-affected regions of Southeast Asia. In fact, the contribution of rice to the daily intake of As might be 25 times higher than that of drinking water [4]. Therefore, reducing the exposure of As through drinking water alone is not enough to combat the plight in As-endemic regions where rice is the major food crop [5].

Rice, the primary staple of almost half of the world’s population, alone meets one-fourth of the global calorific need [6]. Over 90% of the world’s rice is produced and consumed in Asian countries, where it is also an important commodity to export and earn foreign exchange. Unfortunately, unlike other major crops, rice is an inherently efficient As accumulator owing to the presence of highly efficient silicic acid transporters in its roots [7]. Added to this, the conventional anaerobic flooded cultivation of rice and use of As-laden groundwater for irrigation exacerbate the situation by increasing the mobility and total load of As in soil [8]. The presence of As in rice not only challenges India—the world’s largest rice exporter—but, more importantly, exposes over half of its huge population to serious health threats. West Bengal, the largest rice producing state in India, is heavily devastated by the chronic and acute health hazards associated with As. More than 26 million people across 2600 villages in this state ingest this toxicant from food and water on a regular basis [9]. Residents of rural Bengal consume, on average, 450 g rice per capita per day to meet 73% of their daily calorific needs which, in endemic regions, is found laced with As, sometimes at levels as high as 0.961 mg kg^−1^ [10]. 

Among several strategies proposed for reducing the As load in rice, the most sought-after ones are water management, application of organic and inorganic amendments, and selection and breeding of low-As-accumulating cultivars. Although water management in the form of alternate wetting and drying shows promising results in reducing the As content in rice [11], the apparent policy constraints and social limitations have stifled its diffusion and adoption in BDP [12]. Organic amendments, on the other hand, show an inconsistent influence on the accumulation of As by rice. There are plenty of reports that reveal the enhanced mobility of As in soil after the application of organic amendments due to reduction in the redox potential and dissolution of Fe-oxyhydroxides [13]. Studies that report the success of inorganic chemical compounds (containing N, P, S, Fe, etc.) in reducing As accumulation in rice do not put much focus on the practical realizability of the doses employed and cost effectiveness of the amendments used. These aspects must be considered for the resource-constrained farming communities of BDP [14]. Silicon (Si) is beneficial for the growth and production of paddy rice. The capacity of Si, as a chemical amendment, to alleviate the stress of pollutant elements derives from its ability to moderate soil pH, change elemental speciation, form coprecipitates, compete for entering the plant body, and bring about defensive physiological changes in plants [15]. 

The application of Si is found to be useful in reducing As uptake by rice [16,17]. However, such studies have been conducted mostly with very high rates of applied silicate, e.g., 70 t ha^−1^ and are surprisingly scarce in BDP—the global hotspot of As poisoning. The only study, to the best of our knowledge, which used relatively smaller doses (0.093–0.75 g Si kg^−1^ soil) of sodium silicate showed no decline in As uptake by paddy rice, rather it reported the growth inhibition of paddy seedlings that were grown on contaminated acid soils [18]. It seems that the different outcomes of Si supplementation in As-contaminated soils is a function of the nature and properties of the soil itself. Hence, fixing practically feasible doses of Si as an amendment against As hazard in rice grown on contaminated soil of West Bengal, India, is very much needed. The development of low-As-accumulating varieties could have been the most feasible strategy to alleviate the rice As hazard only if it was not so time consuming (5–10 years) to introduce a new trait to a variety. The screening of already popular rice cultivars with a low grain As content may be a worthwhile alternative. Most such cultivar-oriented studies conducted in BDP so far have been concentrated in Bangladesh [19,20]. Separate efforts are required to find out which popular cultivars accumulate less As in grain when grown on naturally contaminated soil of West Bengal.

It has been proven that Si-based amendments have the potential to reduce the As load in rice grain. However, the efficacy of the same has been tested neither in Bangladesh nor in West Bengal, India, despite the dire mass arsenic poisoning that is occurring in BDP. This study is required to understand the dose and extent to which Si can provide safeguards against the rice As hazard when applied to the cultivars popularly grown on naturally As-contaminated soil of West Bengal, India. The resource-constrained farmers of West Bengal, India, need an economically feasible yet easy-to-adopt solution (unlike accepting a newly developed cultivar that is low-arsenic-accumulating or following a revised water regime) to this seemingly never-ending problem. In this study, we integrated two such strategies and applied them to the soil of West Bengal that has been polluted by arsenic for a long time. The root exudates significantly influence the bio-geochemical cycling of elements in the rhizosphere [21].

The effect of the composition as well as the magnitude of the root exudates on the accumulation of As by rice plants has not as been studied yet. Examining the behavior of organic acids secreted by the roots of different rice cultivars in response to applied As will definitely open up a new area of interest. In connection therewith, the present investigation was undertaken with the following objectives: (i) to screen four rice cultivars commonly grown in As-endemic areas of India by determining the As uptake in different plant parts; (ii) to assess the efficacy of applied silicate on reducing the As uptake by different rice cultivars; (iii) to examine the effect of As on the release of organic acid from the roots of different rice cultivars in solution culture; (iv) to assess the efficacy of integrated (amendment plus cultivar-oriented) remediation option in reducing the health risks posed by the intake of As through rice.

## 2. Materials and Methods

### 2.1. Soil Sample Collection and Characterization

One bulk sample of surface (0–15 cm) soil was collected from the geogenically arsenic (As)-affected area of Mitrapur village (22.9981° N, 88.6121° E; 8.8 m above MSL), Haringhata block, Nadia District of West Bengal, India. The collected soil sample was dried under shade, crushed, and then passed through a 2 mm sieve. The processed soil sample was analyzed for pH (soil/water, 1:2), organic carbon, electrical conductivity, and cation exchange capacity following the procedures used by Jackson (1973) [22]. The soil textural composition was determined by the hydrometer method [23]. For extractable As in soil, 0.5 *M* sodium bicarbonate solution (pH 8.5) was used as the extractant [24]. For determining the pseudo-total As, a soil sample was digested in a microwave digester (Multiwave ECO, Anton Paar). For this purpose, 0.2 g of processed soil was digested with 7 mL of concentrated (65%) suprapure HNO_3_ (Merck KGaA, Germany) inside a PTFE-TFM vessel (microwave-assisted extraction). The operational conditions were set to reach a temperature of 180–190 °C with a 25 min ramp time and a hold time of another 25 min to maintain the same temperature. After the extraction was completed, the vessels were cooled down to room temperature, vented, and the lid was opened. The extract was diluted using Milli-Q water and filtered. The concentration of As (both extractable and pseudo-total) was determined by inductively coupled plasma-mass spectrometer (ICP-MS, PerkinElmer NexION 300). The operating parameters of the ICP-MS are presented in Table S1. Table 1 summarizes the physicochemical properties of the initial soil. 

### 2.2. Experiments

A greenhouse pot experiment and a solution culture experiment were conducted simultaneously with four popular rice cultivars, including three dwarf nonaromatic high-yielding varieties (HYVs), viz., IR-36 (V1), Khitish (V2), Satabdi (V3), and one tall indigenous short-grained aromatic variety, Badshabhog (V4). These were obtained from Bidhan Chandra Krishi Viswavidyalaya, Mohanpur, in West Bengal, India. The pot experiment was conducted to assess the variability among the cultivars in As uptake, as influenced by the silicate supplement. The purpose of the solution culture experiment was to, firstly, assess the impact of As on rice-root-secreted organic acid content and, secondly, to see how it corroborates the As uptake result of the pot experiment. Each treatment was performed in triplicate in both the experiments.

#### 2.2.1. Pot Experiment

Sodium metasilicate (Na_2_SiO_3_) was added to the soils in the pot at rates of 0 (S0), 250 (S1), and 500 (S2) mg kg^−1^ soil, which were roughly equivalent to 0, 500, and 1000 kg Na_2_SiO_3_ or 0, 114.7, and 229.3 kg Si ha^−1^. Each standard rounded pot was filled with four kg of soil. Nitrogen, P, and K fertilizers were also applied as per the recommended rates. All the fertilizers including Na_2_SiO_3_ were applied in solution form. The soil in each pot was irrigated and kept at saturation for seven days for attaining equilibration before sowing. The subsequent thinning (to 5 plants per pot), top-dressing, and other intercultural operations were carried out accordingly. The water level was maintained at 3 cm above the soil surface until harvest. The mature plants were harvested and subsequently separated into grain (unpolished), husk, and straw. The harvested plants were washed and oven-dried at 60 ± 5 °C. On attaining a constant weight, the straw, husk, and grain samples were ground to powder for subsequent chemical analyses. The post-harvest soil samples were collected, dried under shade, ground, and passed through a 2 mm sieve to determine the extractable As content.

#### 2.2.2. Solution Culture Experiment

On the 7th day after sowing, two well-germinated seedlings of each rice cultivar were transferred to amber-colored conical flasks containing 300 mL of modified Hoagland solution [25], with two doses of As, viz., 0 and 100 µg L^−1^. The concentration of As in solution culture (i.e., 100 µg L^−1^) was selected so that it closely matched the intensity of As in the soil solution of contaminated soils [26]. The volume of the solution was maintained on a regular basis. The plants were raised in the solution for 40 days. Two samplings of rooting solution were conducted on the 20th and 40th days after transplanting. The nutrient solution was changed regularly to check the microbial growth. The samples were stored at −4 °C. After the second sampling, the plants were taken out, acid-washed, separated into roots and shoots, oven-dried, weighed, ground, and analyzed.

### 2.3. Plant Analysis

The plant samples (obtained from both the soil and solution culture experiments) were digested by a microwave digester using concentrated (65%) suprapure nitric acid (Merck KGaA, Germany) (microwave-assisted digestion) [27]. The arsenic in the digest was determined by ICP-MS (PerkinElmer NexION 300). SRM 1573a (tomato leaves) from NIST was used to validate the ICP-MS results. The average recovery percentage was 95.3 ± 4.01% for As. 

### 2.4. Soil Analysis

The extractable As content in the post-harvest soil was determined following the procedure outlined by Golui et al. (2017a) [28]. The arsenic concentration in the soil extract was measured by ICP-MS (PerkinElmer NexION 300). We spiked the arsenic standard (5 mg kg^−1^) in a soil, and the observed recovery was 91.3 ± 3.7%.

### 2.5. Computation of the Translocation Coefficients

The relative As translocation efficiency of different rice cultivars was expressed in terms of the translocation coefficient from husk to grain (TC_hg_), as well as from straw to husk (TC_sh_), using the following formulae:

TC_hg_ = (As content in brown rice/As content in husk) (1)

TC_sh_ = (As content in husk/As content in straw) (2)

To obtain a clearer picture, the translocation coefficient from root to shoot (TC_rs_) was calculated from the data obtained from solution culture experiment using the following formula:

TC_rs_ = (As content in shoot/As content in root) (3)

### 2.6. Characterization of the Rooting Solution

The concentration of total weak acid was determined by conductometric titration. At first, 50 mL of the rooting solution was repeatedly passed through 50 g of acid-free cation exchange resin (CER) (AMBERLITE IR-120 H^+^) until the eluate attained a constant pH. The titration was carried out on a known volume of the final eluate with 0.05 *N* ammonium hydroxide (NH_4_OH) as the titrant. The pre-standardized NH_4_OH was added in a small increment, preferably 0.1 mL. The electrical conductivity of the titrant was measured after each incremental addition, and titration was continued until the EC attained a constant value. The concentration of the total weak acid was calculated using the following formula:

Concentration of total weak acid (m*N*) = (v × *N* × 1000)/V (4)

where v = volume of the NH_4_OH consumed to neutralize weak acid (mL), *N* = normality of the NH_4_OH solution, and V = volume of the rooting solution taken (20 mL).

Mainly, malic, succinic, citric, and lactic acids have been reported as important components in the root-exudate of rice [29]. In the present investigation, these acids were quantified by high-performance liquid chromatography (1200 Infinity; Agilent Technologies, Santa Clara, CA, USA) following the procedure of Krishnapriya and Pandey (2016) [30]. 

### 2.7. Human Health Hazard for As Intake through Rice Consumption

The hazard quotient (HQ) was calculated for assessing the potential noncarcinogenic risk brought about through the intake of As via contaminated rice [31]. The hazard quotient is expressed as the ratio of the average daily dose (ADD; mg kg^−1^ d^−1^) of As to that of its reference dose (RfD; mg kg^−1^ d^−1^). The reference dose is the maximum tolerable daily intake of As that does not result in any detrimental effect on health.

HQ = ADD/RfD(5)

The daily intake of rice grain by adult humans in the study area was taken as 0.45 kg day^−1^ [32]. The average adult body weight was assumed to be 70 kg.

HQ = (M_grain_ × W)/(RfD × 70) (6)

where M_grain_ = grain inorganic As content (mg kg^−1^) of the rice grown in contaminated soil, and W = the daily intake of rice grain (0.45 kg). On average, 73% of the As present in rice was assumed to be inorganic [33]. The hazard quotient for children was calculated by assuming the daily intake and body weight as 0.20 kg day^−1^ [34] and 30 kg [35], respectively.

The cancer risk (CR) was calculated by multiplying the ADD with the cancer slope factor (CSF) [34].

CR = ADD × CSF(7)

where the CSF of arsenic is 1.5 (mg kg (BW)^−1^ day^−1^)^−1^ [36].

### 2.8. Statistical Analysis

The analysis of variance (ANOVA) method, as suggested by Snedecor and Cochran (1967) [37], was followed as per the objectives of the investigation in RStudio 1.3.1093 [38] using the “doebioresearch” package [39]. For pair-wise comparison of the means, Duncan’s multiple range test (DMRT) was implemented after the significant overall F test using the same package in RStudio.

## 3. Results and discussion

### 3.1. Pot Culture Experiment

#### 3.1.1. Effect of the Applied Silicate on the Extractable Arsenic (As) in Soil 

Upon application of the silicate, the Olsen extractable As content in the post-harvest soil significantly increased from 3.10 (in S0) to 3.97 (in S1) and 3.89 mg kg^−1^ (in S2), indicating that S1 and S2 were statistically at par (Table 2). Li et al. (2018) [16] also reported a 1.5-fold increase in the As concentration in soil solution when the soil was amended with silica gel at the rate of (@) 20 g kg^−1^. Arsenic remains in soil in different chemical forms owing to its different oxidation states. In reduced soil, the dominant species of As are arsenous acid (H_3_As(III)O_3_^0^) and different arsenite oxyanions (H_2_As(III)O_3_^−^, HAs(III)O_3_^2−^). Applied Na_2_SiO_3_ in soil undergoes transformation, forming silicate anions depending on the soil pH. The arsenite oxyanions, being weaker ligands, can easily be replaced by relatively stronger ligands such as silicate [40], which is reflected by the elevated level of As in the soils of the Si-amended pots. 

#### 3.1.2. Effect of the Cultivar and Applied Silicate on the As Content 

On an average, in the control, the largest amount of As was found in straw (3.93 mg kg^−1^) followed by husk (0.69 mg kg^−1^) and grain (brown rice) (0.29 mg kg^−1^). In the absence of applied silicate, the highest As content in the brown rice was recorded with Satabdi (0.4 mg kg^−1^), followed by IR-36 (0.36 mg kg^−1^) and Khitish (0.28 mg kg^−1^), whereas, the lowest As content was recorded with Badshabhog (0.11 mg kg^−1^) (Table 2). As per the Codex Committee on Contaminants in Food (CCCF) recommended safe limit of As (0.2 mg kg^−1^) in rice grain, all these cultivars, except Badshabhog, were deemed unfit for human consumption. On average, all four rice cultivars accumulated almost similar amounts of As in the husk. Based on the straw As content, the cultivars can be arranged in the following order: Khitish (6.01 mg kg^−1^) > IR-36 (3.80 mg kg^−1^) = Satabdi (3.37 mg kg^−1^) > Badshabhog (2.52 mg kg^−1^).

The nonaromatic high-yielding cultivars accumulated remarkably higher amounts of As in grain (2.54- to 3.63-fold) as compared to the aromatic genotype, Badshabhog. Sandhi et al. (2017) [41] did find that aromatic rice cultivars accumulate less As than nonaromatic ones. The elevated expression of *OsABCC1*, a gene, apparently among many others, responsible for detoxification and reduced grain allocation of As, was noted in traditional aromatic rice cultivars in [42]. The symplastic discontinuity encountered by the metalloid during its journey from husk to grain has also been suspected as one of the mechanisms barring the entry of As into rice endosperm [43]. Badshabhog accumulated the least As in straw. The low accumulation of As in rice cultivars has also been attributed to anatomical characters, such as a narrower leaf blade and fewer vascular bundles in the leaf compared to that in high-yielding rice cultivars. Apart from these abovementioned genetic and anatomical justifications, this study, as shown in Section 3.2.2, for the first time, sheds light on what role root-secreted acids play in determining the As acquisition capacity of rice cultivars. Badshabhog is an indigenous aromatic rice cultivar with a much lower As content and higher market value than the remaining three high-yielding varieties.

On an average, the application of Na_2_SiO_3_ @ 250 (S1) and 500 (S2) mg kg^−1^ reduced the As content in grain to the tune of 17.2 and 27.6%, respectively (Table 2). When compared to the control, the grain As content was significantly reduced in all cultivars under S1 and S2. However, the effects of S2 and S1 were statistically at par in all four cultivars. The grain As content of Badshabhog was reduced to 72.7% under S1 and S2, bringing down the absolute value to 0.03 mg kg^−1^. In the case of Khitish, S2 resulted in a 39.3% abatement in the grain As content, scaling it down below the CCCF recommended safe limit (0.2 mg kg^−1^) and subsequently making it safe for human consumption. With respect to control, 26.1 and 17.3% reduction in the As content were obtained in husk under S1 and S2, respectively, with the corresponding reduction in the straw were 21.1 and 18.8%. In spite of increasing the availability of As in the soil, the application of silicate could reduce the As content in the grain, husk, and straw of paddy. Under submerged paddy soils (having Eh = 0 to 0.1V and a pH ranging from 6 to 8), the major stable form of As is undissociated arsenous acid (H_3_As(III)O_3_^0^; pK = 9.2) [44]. Because arsenous acid and silicic acid demonstrate structural similarity and both have high pK_a1_ values (9.0–9.3), arsenite competes with silicic acid for entry and further translocation via Si-transport pathways in paddy (Lsi1 and Lsi2) [45]. The negative effect of the applied silicate on the uptake of As by plants can be explained on the basis of competition between silicic acid and arsenious acid under elevated concentrations of the former in soil solution upon external application of Na_2_SiO_3_. 

Conversely, the positive effect of the silicate application on the As content in the seedlings of rice was earlier reported [18], and it may have resulted from the fact that application of silica gel in a much higher concentration brought significantly more As in the soil solution through ligand exchange. In addition, the higher solubility of Na_2_SiO_3_ (compared to silica gel) helped inhibit the uptake and translocation of As [46]. For the sustainable production of rice and to reduce the load of toxic As in its consumable parts (i.e., grain for humans and straw for animals), it is important to determine the dose of the silicate critically in order to maintain the balance between the competition of Si and As for both soil exchange sites and plant cells’ transporters. In a few earlier studies, the negative effect of Si on As absorption by paddy rice was reported for a much higher rate (10 to 20 t ha^−1^) of applied Si-based amendments [16,47]. In the present investigation, much smaller practically realizable rates of Si were found to be effective in reducing the As content in rice grain and other tissues when applied in the contaminated soil of West Bengal, India. 

#### 3.1.3. Translocation Coefficient of As in Plant Parts

**Figure 1:**
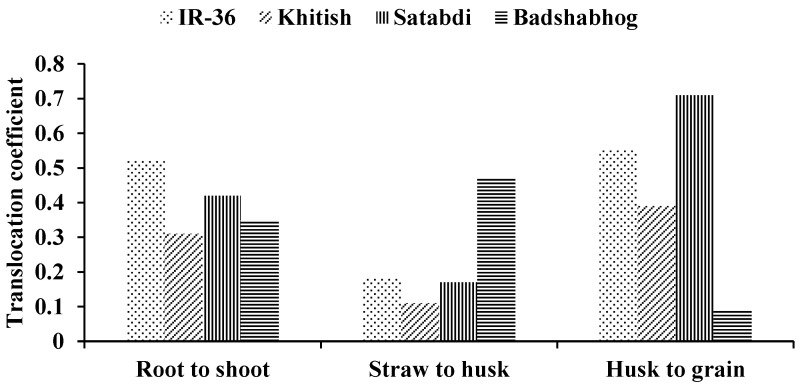
Translocation coefficients for the root to shoot, straw to husk, and husk to grain translocation of As in the different rice cultivars.

On an average, the translocation coefficient of As from root to shoot (TC_rs_) ranged from 0.31 (Khitish) to 0.52 (IR-36). The mean translocation coefficient of As from straw to husk (TC_sh_) ranged from 0.11 (Khitish) to 0.47 (Badshabhog). The highest translocation coefficient of As from husk to grain (TC_hg_) was associated with Satabdi, followed by IR-36, Khitish, and Badshabhog; the corresponding values of TC_hg_ were 0.71, 0.55, 0.39, and 0.09. The application of silicate did not indicate a significant effect on TC_sh_ or TC_hg_. The variation in the translocation coefficients among the cultivars is depicted in Figure 1.

This clearly shows that the substantial share of the total As entering the plant accumulated in the root system. The findings reported in the present investigation agreed with those of previous studies that the accumulation of As in different parts of rice follows the order root >> shoot > husk > grain. Rahman et al. (2007b) [48] reported that the root, shoot, and grain of the rice cultivar BRR1 dhan28 contained, respectively, 96, 3, and 1% of the total uptake of As. After absorption, arsenate is reduced to arsenite inside plant with the help of arsenate reductase enzyme [49]. Subsequently, arsenite forms complexes with thiol-rich peptides leading to its detoxification inside the plant root [50]. The formation of organo-As complexes and the subsequent storage in vacuoles of root cells result in a relatively much greater accumulation of As in plant root [49]. IR-36, being a larger accumulator of As in grain, showed the highest TC_rs_, which means that the mechanisms for As sequestration in root are weaker in IR-36 than in the remaining three low-As-accumulating cultivars. Although Badshabhog showed the most efficient translocation from straw to husk, this cultivar was the least efficient translocator of As from husk to grain. This outcome indicates the possibility of the existence of larger symplastic discontinuity in Badshabhog. In most previous studies, TC_hg_ was not assessed separately, and the translocation of As from shoot to direct grain was examined. This suggests the possibility of the barrier effect of husk against As [51]. While Bilo et al. (2015) [52] suspected the probable straining effect of rice husk for heavy metals, the exact mechanism(s) for As is/are yet to be explained. Cultivating varieties with lower husk to grain translocation coefficients can be a cheap and effective way of harnessing nature’s mechanism for eliminating this toxicant from the human food chain.

### 3.2. Solution Culture Experiment

#### 3.2.1. Root and Shoot Arsenic Content of the Cultivars

**Figure 2:**
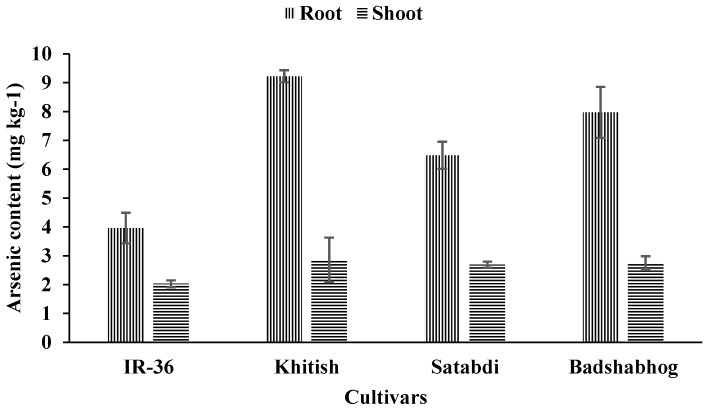
Arsenic content in the root and shoot of the different rice cultivars grown in Hoagland solution containing 100 μg L^−1^ As.

On an average, the As content in rice root, under 100 μg L^−1^ As in solution, was 6.90 mg/kg. The highest root As content was recorded in Khitish (9.22 mg kg^−1^), followed by Badshabhog (7.97 mg kg^−1^) and Satabdi (6.48 mg kg^−1^) (Figure 2). IR-36 showed the lowest concentration (3.96 mg kg^−1^) of As in its root. The storage of As in root is one of the mechanisms by which plants avoid the translocation of higher amounts of As in grain or other aboveground parts [8]. On the other hand, the shoot-As-content, under 100 μg L^−1^ As in solution, ranged over 2.03–2.86 mg kg^−1^ but did not vary significantly among the cultivars (Figure 2).

#### 3.2.2. Effect of As on the Total Weak Acid Secretion by the Roots of Different Rice Cultivars

The results of the total concentration of weak acid in solution were significantly affected by the rice cultivars and As (Table 3). On average, the concentration of the total weak acid in rooting solution rose significantly by 34% (from 0.47 to 0.63 m*N*) in response to the increase in the concentration of As in solution from 0 to 100 µg L^−1^. The highest concentration of total weak acid was secreted by IR-36 (0.82 m*N*), whereas Satabdi (0.44 m*N*) and Badshabhog (0.36 m*N*) released the smallest amounts of total weak acids in solution culture.

No direct evidence could be cited to support such a result, since no research related to assessing the impact of As on the secretion of organic acids by rice roots could be traced in the literature. The organic acid exudation by plant roots is stimulated by external stress. Montiel-Rozas et al. (2016) [53] reported the enhanced release of low molecular weight organic acids by other plant species under the stress of metals (Cd, Cu, and Zn), which may be considered as plants’ defense mechanism to mitigate metal toxicity through chelation. The hydroxyl, phenolic, and carboxylic groups present in organic compounds can form complexes with As (both arsenite and arsenate) via different mechanisms, such as ligand exchange and hydrogen bonding [54,55]. The root exudate of *Pteris vittata* L. was reported to comprise formic, acetic, malic, oxalic, succinic, and citric acids [56]. Of these acids, the concentration of malic, oxalic, and succinic acids was significantly elevated due to the applied As. In contrast, no such increase in the release of the mentioned acids was reported for non-hyper accumulating plant (Boston Fern, *Nephrolepis exaltata* L.) [57].

In hyperaccumulating *Pteris vittata* L., the secretion of higher amounts of low molecular weight organic acids leads to the dissolution of As-containing minerals, consequently enhancing the intensity of As in soil solution [56]. On an average, based on the secretion of total weak acids, the rice cultivars could be arranged in the following order: IR-36 > Khitish > Satabdi = Badshabhog. The positive correlation (r = 0.91) between the grain As content and the concentration of root-secreted total weak acid by the rice cultivars in the presence of As in solution suggests that the lower secretion of total weak acid was one of the important factors that made Badshabhog an inefficient As accumulator compared to the high-yielding (and higher As-accumulating) varieties. It has been established that in As-enriched soils silicon-based fertilizers can significantly increase the yield of rice [58] by bringing about numerous positive changes in the agro-ecosystem functions [59,60]. The information generated in this study may lead to a new path for making progress in this research area.

#### 3.2.3. Effect of As on Specific Organic Acids as Secreted by the Roots of the Different Rice Cultivars

**Figure 3:**
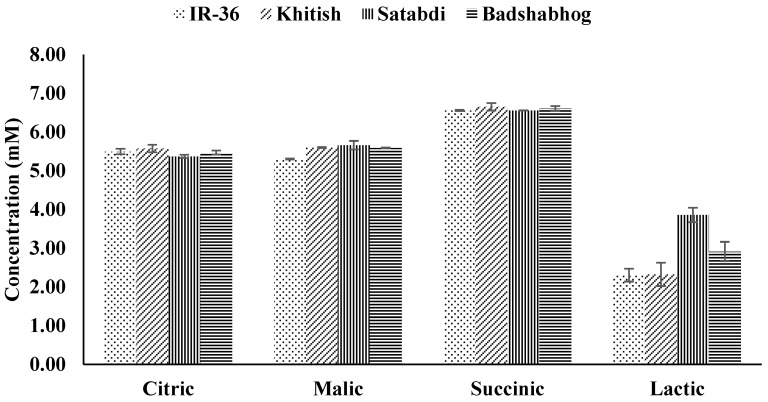
Concentration of the different organic acids secreted by the roots of the different rice cultivars in solution culture.

In this study, the type of acid showing the highest secretion was succinic (6.59 m*M*), followed by malic (5.54 m*M*), citric (5.48 m*M*), and lactic acid (2.85 m*M*). Aulakh et al. (2001) [29] also indicated that rice cultivars released the least amount of lactic acid among malic, tartaric, succinic, citric, and lactic acids in root exudate. Only lactic acid differed significantly among cultivars. The highest amount (i.e., 3.86 m*M*) of lactic acid was secreted by Satabdi, and this was followed by Badshabhog (2.91 m*M*), whereas IR-36 (2.30 m*M*) and Khitish (2.32 m*M*) secreted similar amounts of lactic acid in solution. None of the acids were significantly affected by the concentration of added As or sampling time. The concentration of different organic acids secreted by the roots of different rice cultivars in solution culture are presented in Figure 3. Further detailed research including other organic acids is required.

### 3.3. Risk Assessment for As Intake by Humans through Rice

The maximum allowable limit (MAL) of As in rice grain has been proposed and revised by many global bodies, such as the WHO (1 mg kg^−1^), EU (0.5 mg kg^−1^), and USDA (0.15 mg kg^−1^) [39]. Recently, in the Eighth Session of CCCF, it has been proposed to fix the MAL of inorganic As load in polished rice at 0.2 mg kg^−1^ [61]. Although popularly used in most parts of the world, such generalized MAL in food materials has no real rationale in the assessment of health risk in view of the fact that the intake of As through a particular food material varies, with widely varying dietary patterns from region to region. This is why the MAL of As in rice grain requires such frequent revisions. It makes more sense if the health risk to toxicants, occurring through the consumption of contaminated food crops, is expressed in terms of a hazard quotient (HQ).

**Figure 4:**
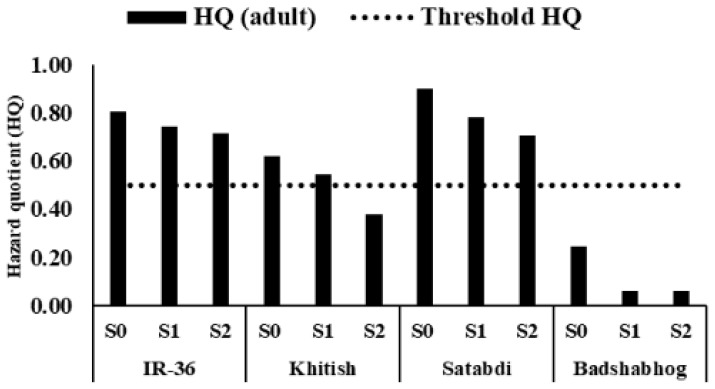
Hazard quotient values for arsenic in different rice cultivars under silicate treatments (sodium metasilicate (Na_2_SiO_3_) was added to the soils in pot at the rates of 0 (S0), 250 (S1), and 500 (S2) mg kg^−1^ soil).

In case of adults, the HQ for As intake through rice ranged from 0.06 to 0.90 across the treatments (Figure 4). Values of an HQ ≥ 1 render the corresponding food material unsafe for human consumption. In this case, the use of such a higher safe limit (i.e., 1) for the HQ appears to be quite unjustifiable, as rice is not the only route for As to enter the human body in the study area. Drinking water, other food materials, dust inhalation, direct ingestion of soil, etc., also contribute significantly to the total intake of As by human. Studies conducted throughout BDP have documented the contribution of rice in human As intake to be approximately 50% [62,63]. Therefore, the allowable safe limit for an HQ of 0.5 is considered proper for rice [64]. A perusal of the data indicates that the HQ values exceeded 0.5 for IR-36 and Satabdi in all Si treatments. In the case of Khitish, the HQ values exceeded 0.5 when Si was applied @ 0 and 250 mg kg^−1^, but it was within 0.5 when Si was applied @ 500 mg kg^−1^. Only for Badshabhog, the HQ values were below 0.5 under all levels of applied silicate. This cultivar showed a conspicuously small HQ (0.06) when silicate was applied.

In all the cultivars, the HQ decreased with a concomitant increase in the dose of silicate, which indicates the effectiveness of silicate in reducing the As load from the rice-based food chain. Once any toxic metal or metalloid gets into the plough layer of soil, its removal becomes very difficult, and it starts adversely affecting the sustainable production of crops by taking a toll on the health and livelihoods of the consumers-cum-residents. The outcome of the present study has the potential to mitigate the ongoing As menace currently prevailing in the rice growing belt of As-polluted BDP. This study estimated the As-health-risk for children too. The HQ for As intake through rice ranged from 0.06 to 0.93 across the treatments (Figure S1). The HQ in three high-yielding varieties ranged between 0.50 and 0.93. However, the same remained below 0.5 (0.06–0.25) in case of Badshabhog. The carcinogenic risk arising from the dietary intake of As-laced rice grain was assessed for adults and children in terms of incremental lifetime cancer risk (ILCR) (Table S2). In every case, the value was higher compared to the threshold level for cancer risk, which is 1 × 10^−6^ [36]. However, its magnitude decreased when Si was added.

## 4. Conclusions

Large-scale arsenic (As) poisoning, mainly through food chain contamination, is deemed to be a lethal problem in the rice-growing areas of the Bengal Delta Plain (BDP) and other parts of the world. This study proposed a feasible strategy for reducing the bioaccumulation of As in a rice-based food chain using low-As-accumulating rice cultivars and silicate. Badshabhog is an indigenous aromatic rice cultivar with a much smaller accumulation of As in the grain (and a high market value). This underpins the importance of rapidly disappearing indigenous cultivars in strengthening food safety for overall sustainable development and the need for their conservation. The arsenic content of straw, husk, and brown rice was significantly reduced with the applied silicate. Particularly, Khitish was rendered fit for human consumption by applied sodium metasilicate @500 mg kg^−1^, which is approximately equivalent to 1 t ha^−1^. The solution culture study showed that cultivars secreting higher quantities of organic acids accumulated elevated levels of As in grain. This opens up the possibility and practical feasibility of using silicate as a cost-effective amendment for reducing As in the hotspot areas. Furthermore, if found satisfactory in multilocational field trials, the conjoint use of sodium metasilicate (@500 mg kg^−1^) and low-As-accumulating rice cultivars—Badshabhog and Khitish—could emerge as an effective option to limit the dietary intake of As through rice within safe limits and reduce people’s exposure to As.

## Figures and Tables

**Table 1:** Initial characteristics of the experimental soil.

Parameter	Value
pH_1:2_	6.97
EC_1:2_ (dS m^−1^)	0.48
Mechanical composition	
Clay%	42.9
Silt%	9.3
Sand%	47.8
Texture	Sandy clay
Organic carbon (g kg^−1^)	16.0
Cation exchange capacity (cmol(p^+^) kg^−1^)	27.8
Pseudo-total As (mg kg^−1^)	27.7
NaHCO_3_ extractable As (mg kg^−1^)	3.10

**Table 2:** Effect of the sodium metasilicate (Na_2_SiO_3_) addition on extractable As (mg kg^−1^) in soil and As content (mg kg^−1^) in the grain, husk, and straw of different rice cultivars *.

Rice Cultivar (C)	Olsen-Extractable As (mg kg^−1^)				Grain Content				Husk Content				Straw Content			
	Silicon Treatment (Si)			Mean	Silicon Treatment (Si)			Mean	Silicon Treatment (Si)			Mean	Silicon Treatment (Si)			Mean
	S_0_	S_1_	S_2_		S_0_	S_1_	S_2_		S_0_	S_1_	S_2_		S_0_	S_1_	S_2_	
IR-36	3.02	4.07	4.18	3.76	0.36 ^ab^	0.33 ^ab^	0.32 ^abc^	0.34 ^A^	0.63	0.51	0.78	0.63	3.80	3.26	3.32	3.47 ^B^
Khitish	2.35	4.40	3.96	3.57	0.28 ^bc^	0.24 ^cd^	0.17 ^de^	0.23 ^B^	0.81	0.54	0.49	0.61	6.01	4.98	5.47	5.48 ^A^
Satabdi	3.56	3.65	3.86	3.69	0.40 ^a^	0.35 ^ab^	0.32 ^bc^	0.36 ^A^	0.57	0.51	0.49	0.52	3.37	3.38	2.79	3.18 ^B^
Badshabhog	3.45	3.75	3.56	3.59	0.11 ^e^	0.03 ^f^	0.03 ^f^	0.05 ^C^	0.75	0.49	0.52	0.58	2.52	0.80	1.18	1.50 ^C^
Mean	3.10 ^B^	3.97 ^A^	3.89 ^A^		0.29 ^A^	0.24 ^B^	0.21 ^B^		0.69 ^A^	0.51 ^B^	0.57 ^B^		3.93 ^A^	3.10 ^B^	3.19 ^B^	

* The values with completely different superscript letters are significantly different. Lowercase and uppercase superscript letters are used for interaction and main effects, respectively. No superscript letter indicates no significant difference.

**Table 3:** Effect of the cultivars and arsenic on the concentration (m*N*) of total weak acid secreted by rice roots in solution culture as sampled at twenty and forty days after transplanting *.

Rice Cultivar (C)	Time of Sampling (Days after Transplanting) (D)							Concentration of Arsenic in the Solution (µg L^−1^) (As)		
	20			40			Mean			
	As (µg L^−1^)			As (µg L^−1^)						
	0	100	Mean	0	100	Mean		0	100	Mean
IR-36	0.82 ^ab^	0.77 ^ab^	0.79	0.77 ^ab^	0.93 ^a^	0.85	0.82 ^A^	0.79 ^ab^	0.85 ^a^	0.82 ^A^
Khitish	0.35 ^de^	0.73 ^abc^	0.53	0.54 ^bcd^	0.72 ^abc^	0.63	0.58 ^B^	0.53 ^cd^	0.63 ^bc^	0.58 ^B^
Satabdi	0.11 ^e^	0.72 ^abc^	0.42	0.29 ^de^	0.65 ^abc^	0.47	0.44 ^C^	0.20 ^e^	0.68 ^abc^	0.44 ^C^
Badshabhog	0.31 ^de^	0.45 ^cd^	0.38	0.25 ^de^	0.44 ^cd^	0.35	0.36 ^C^	0.35 ^de^	0.37 ^de^	0.36 ^C^
Mean	0.43	0.63	0.53	0.51	0.64	0.57		0.47 ^B^	0.63 ^A^	

* The values with completely different superscript letters are significantly different. Lowercase and uppercase superscript letters are used for interaction and main effects, respectively. No superscript letter indicates no significant difference.

## Data Availability

Not applicable.

## Supplementary Materials

The following supporting information can be downloaded at: https://www.mdpi.com/article/10.3390/toxics11010064/s1, Figure S1: Hazard quotient values for arsenic for children in different rice cultivars under silicate treatments (Sodium metasilicate (Na_2_SiO_3_) was added to the soils in pot at the rates of 0 (S0), 250 (S1) and 500 (S2) mg kg^−1^ soil); Table S1. Operating conditions for ICP-MS (Model- Perkin Elmer, NexIon 300); Table S2. Carcinogenic risk in adult and children through the dietary intake of rice grain.

## References

[1] Islam M., Mostafa M.G. (2021). Influence of chemical fertilizers on arsenic mobilization in the alluvial Bengal delta plain: A critical review. J. Water Supply Res. Technol.-AQUA.

[2] Beyer W.N., Cromartie E.J. (1987). A survey of Pb, Cu, Zn, Cd, Cr, As and Se in earthworms and soil from diverse sites. Environ. Monit. Assess..

[3] World Health Organization (2018). Arsenic. https://www.who.int/news-room/fact-sheets/detail/arsenic.

[4] Liang F., Li Y., Zhang G., Tan M., Lin J., Liu W., Li Y., Lu W. (2010). Total and speciated arsenic levels in rice from China. Food Addit. Contam..

[5] Paul S., Das N., Bhattacharjee P., Banerjee M., Das J.K., Sarma N., Sarkar A., Bandyopadhyay A.K., Sau T.J., Basu S. (2013). Arsenic-induced toxicity and carcinogenicity: A two-wave cross-sectional study in arsenicosis individuals in West Bengal, India. J. Expo. Sci. Environ. Epidemiol..

[6] Zhu C., Kobayashi K., Loladze I., Zhu J., Jiang Q., Xu X., Liu G., Seneweera S., Ebi K.L., Drewnowski A. (2018). Carbon dioxide (CO_2_) levels this century will alter the protein, micronutrients, and vitamin content of rice grains with potential health consequences for the poorest rice-dependent countries. Sci. Adv..

[7] Bhattacharyya S., Chatterjee M., Debnath S., Sarkar S., Mukherjee A., Guha Mazumder D.N., Sarkar S. (2012). Genetics and physiology of elevated arsenic in grain and straw of rice. Arsenic Contamination in Water and Food Chain, Proceedings of the International Workshop on “Arsenic in Food Chain: Cause, Effect and Mitigation”, Kolkata, India, 20 February 2012.

[8] Mitra A., Chatterjee S., Moogouei R., Gupta D. (2017). Arsenic accumulation in rice and probable mitigation approaches: A review. Agronomy.

[9] Basu A., Sen P., Jha A. (2015). Environmental arsenic toxicity in West Bengal, India: A brief policy review. Indian J. Public Health.

[10] Halder D., Biswas A., Šlejkovec Z., Chatterjee D., Nriagu J., Jacks G., Bhattacharya P. (2014). Arsenic species in raw and cooked rice: Implications for human health in rural Bengal. Sci. Total Environ..

[11] Sarkar S., Basu B., Kundu C.K., Patra P.K. (2012). Deficit irrigation: An option to mitigate arsenic load of rice grain in West Bengal, India. Agric. Ecosyst. Environ..

[12] Pandey S., Yadav S., Hellin J., Balié J., Bhandari H., Kumar A., Mondal M.K. (2020). Why technologies often fail to scale: Policy and market failures behind limited scaling of alternate wetting and drying in rice in Bangladesh. Water.

[13] Jin W., Wang Z., Sun Y., Wang Y., Bi C., Zhou L., Zheng X. (2020). Impacts of biochar and silicate fertilizer on arsenic accumulation in rice (*Oryza sativa* L.). Ecotoxicol. Environ. Saf..

[14] Moulick D., Samanta S., Sarkar S., Mukherjee A., Pattnaik B.K., Saha S., Awashi J.P., Bhowmick S., Ghosh D., Samal A.C. (2021). Arsenic contamination, impact and mitigation strategies in rice agro-environment: An inclusive insight. Sci. Total Environ..

[15] Bhat J.A., Shivaraj S.M., Singh P., Navadagi D.B., Tripathi D.K., Dash P.K., Solanke A.U., Sonah H., Deshmukh R. (2019). Role of silicon in mitigation of heavy metal stresses in crop plants. Plants.

[16] Li G., Zheng M.Z., Tang J.F., Shim H., Cai C. (2018). Effect of silicon on arsenic concentration and speciation in different rice tissues. Pedosphere.

[17] Islam S., Rahman M.M., Naidu R. (2019). Impact of water and fertilizer management on arsenic bioaccumulation and speciation in rice plants grown under greenhouse conditions. Chemosphere.

[18] Lee C.H., Huang H.H., Syu C.H., Lin T.H., Lee D.Y. (2014). Increase of arsenic release and phytotoxicity to rice seedlings in arsenic-contaminated paddy soils by Si fertilizer application. J. Hazard. Mater..

[19] Rahman M.A., Hasegawa H., Rahman M.M., Islam M.N., Miah M.A.M., Tasmin A. (2007). Arsenic accumulation in rice (*Oryza sativa* L.) varieties of Bangladesh: A glass house study. Water Air Soil Pollut..

[20] Norton G.J., Islam M.R., Deacon C.M., Zhao F.J., Stroud J.L., McGrath S.P., Islam S., Jahiruddin M., Feldmann J., Price A.H. (2009). Identification of low inorganic and total grain arsenic rice cultivars from Bangladesh. Environ. Sci. Technol..

[21] Narula N., Kothe E., Behl R.K. (2012). Role of root exudates in plant-microbe interactions. J. Appl. Bot. Food Qual..

[22] Jackson M.L. (1973). Soil Chemical Analysis.

[23] Bouyoucos G.J. (1962). Hydrometer method improved for making particle size analyses of soils. Agron. J..

[24] Sparks D.L. (2003). Environmental Soil Chemistry.

[25] Hoagland D.R., Arnon D.I. (1950). The water-culture method for growing plants without soil. Circ. Calif. Agric. Exp. Stn..

[26] Fitz W.J., Wenzel W.W. (2002). Arsenic transformations in the soil–rhizosphere–plant system: Fundamentals and potential application to phytoremediation. J. Biotechnol..

[27] Meena R., Datta S.P., Golui D., Dwivedi B.S., Meena M.C. (2016). Long-term impact of sewage irrigation on soil properties and assessing risk in relation to transfer of metals to human food chain. Environ. Sci. Pollut. Res..

[28] Golui D., Datta S.P., Meena M.C., Shukla A.K., Datta S.P., Meena M.C., Dwivedi B.S., Shukla A.K. (2017). Determination of arsenic in water, soil, plant and biomarker. Manual on Advance Techniques for Analysis of Nutrients and Pollutant Elements in Soil, Plant and Human.

[29] Aulakh M.S., Wassmann R., Bueno C., Kreuzwieser J., Rennenberg H. (2001). Characterization of root exudates at different growth stages of ten rice (*Oryza sativa* L.) cultivars. Plant Biol..

[30] Krishnapriya V., Pandey R. (2016). Root exudation index: Screening organic acid exudation and phosphorus acquisition efficiency in soybean genotypes. Crop Pasture Sci..

[31] US EPA (2001). Baseline Human Health Risk Assessment Vasquez Boulevard and I-70 Superfund Site, Denver CO. http://www.epa.gov/region8/superfund/sites/VB-170-Risk.pdf.

[32] Biswas A., Deb D., Ghose A., Du Laing G., De Neve J., Santra S.C., Guha Mazumder D.N. (2014). Dietary arsenic consumption and urine arsenic in an endemic population: Response to improvement of drinking water quality in a 2-year consecutive study. Environ. Sci. Pollut. Res..

[33] Rahman M.M., Asaduzzaman M., Naidu R. (2011). Arsenic exposure from rice and water sources in the Noakhali district of Bangladesh. Water Qual. Expo. Health.

[34] Das A., Joardar M., Chowdhury N.R., Mridha D., De A., Majumder S., Das J., Majumdar K.K., Roychowdhury T. (2022). Significance of the prime factors regulating arsenic toxicity and associated health risk: A hypothesis-based investigation in a critically exposed population of West Bengal, India. Environ. Geochem. Health.

[35] Joardar M., Das A., Chowdhury N.R., Mridha D., De A., Majumdar K.K., Roychowdhury T. (2021). Health effect and risk assessment of the populations exposed to different arsenic levels in drinking water and foodstuffs from four villages in arsenic endemic Gaighata block, West Bengal, India. Environ. Geochem. Health.

[36] US EPA 2005. Guidelines for Carcinogen Risk Assessment. Risk Assessment Forum. United States Environmental Protection Agency, Washington, D.C. EPA/630/P-03/ 001F. https://www.epa.gov/sites/default/files/2013-09/documents/cancer_guidelines_final_3-25-05.pdf.

[37] Snedecor G.W., Cochran W.G. (1967). Statistical Methods.

[38] R Core Team (2020). R: A Language and Environment for Statistical Computing.

[39] Popat R., Banakara K., Doebioresearch: Analysis of Design of Experiments for Biological Research (2020). R Package (Version 0.1.0). https://CRAN.R-project.org/package=doebioresearch.

[40] Fendorf S., Kocar B.D. (2009). Biogeochemical processes controlling the fate and transport of arsenic: Implications for south and southeast Asia. Adv. Agron..

[41] Sandhi A., Greger M., Landberg T., Jacks G., Bhattacharya P. (2017). Arsenic concentrations in local aromatic and high-yielding hybrid rice cultivars and the potential health risk: A study in an arsenic hotspot. Environ. Monit. Assess..

[42] Shri M., Singh P.K., Kidwai M., Gautam N., Dubey S., Verma G., Chakrabarty D. (2019). Recent advances in arsenic metabolism in plants: Current status, challenges and highlighted biotechnological intervention to reduce grain arsenic in rice. Metallomics.

[43] Meharg A.A., Zhao F.J. (2012). Arsenic and Rice.

[44] Kumarathilaka P., Seneweera S., Meharg A., Bundschuh J. (2018). Arsenic speciation dynamics in paddy rice soil-water environment: Sources, physico-chemical, and biological factors-A review. Water Res..

[45] Ma J.F., Yamaji N. (2006). Silicon uptake and accumulation in higher plants. Trends Plant Sci..

[46] Seyfferth A.L., Fendorf S. (2012). Silicate mineral impacts on the uptake and storage of arsenic and plant nutrients in rice (*Oryza sativa* L.). Environ. Sci. Technol..

[47] Fleck A.T., Mattusch J., Schenk M.K. (2013). Silicon decreases the arsenic level in rice grain by limiting arsenite transport. J. Plant. Nutr. Soil Sci..

[48] Rahman M.A., Hasegawa H., Rahman M.M., Rahman M.A., Miah M.A.M. (2007). Accumulation of arsenic in tissues of rice plant (*Oryza sativa* L.) and its distribution in fractions of rice grain. Chemosphere.

[49] Abbas G., Murtaza B., Bibi I., Shahid M., Niazi N.K., Khan M.I., Amjad M., Hussain M. (2018). Arsenic uptake, toxicity, detoxification and speciation in plants: Physiological, biochemical and molecular aspects. Int. J. Environ. Res. Public Health.

[50] Liu G., Cai Y. (2010). Complexation of arsenite with dissolved organic matter: Conditional distribution coefficients and apparent stability constants. Chemosphere.

[51] Chou M.L., Jean J.S., Sun G.X., Hseu Z.Y., Yang C.M., Das S., Teng J.H. (2014). Distribution and accumulation of arsenic in rice plants grown in arsenic-rich agricultural soil. Agron. J..

[52] Bilo F., Lodolo M., Borgese L., Bosio A., Benassi L., Depero L.E., Bontempi E. (2015). Evaluation of heavy metals contamination from environment to food matrix by TXRF: The case of rice and rice husk. J. Chem..

[53] Montiel-Rozas M.M., Madejón E., Madejón P. (2016). Effect of heavy metals and organic matter on root exudates (low molecular weight organic acids) of herbaceous species: An assessment in sand and soil conditions under different levels of contamination. Environ. Pollut..

[54] Inam M.A., Khan R., Akram M., Khan S., Park D.R., Yeom I.T. (2019). Interaction of arsenic species with organic ligands: Competitive removal from water by coagulation-flocculation-sedimentation (C/F/S). Molecules.

[55] Mandal J., Golui D., Raj A., Ganguly P. (2019). Risk assessment of arsenic in wheat and maize grown in organic matter amended soils of Indo-Gangetic plain of Bihar, India. Soil Sediment Contam..

[56] Das S., Chou M.L., Jean J.S., Yang H.J., Kim P.J. (2017). Arsenic-enrichment enhanced root exudates and altered rhizosphere microbial communities and activities in hyperaccumulator *Pteris vittata*. J. Hazard. Mater..

[57] Tu S., Ma L., Luongo T. (2004). Root exudates and arsenic accumulation in arsenic hyperaccumulating *Pteris vittata* and non-hyperaccumulating *Nephrolepis exaltata*. Plant Soil.

[58] Roy A., Datta S.P., Barman M., Bhattacharyya S., Meena M.C., Golui D., Trivedi V.K., Chakraborty R. (2020). Biomass yield of rice (*Oryza sativa*) cultivars as affected by applied silicate in an Inceptisol. Indian J. Agric. Sci..

[59] Das S., Kim G.W., Lee J.G., Bhuiyan M.S.I., Kim P.J. (2021). Silicate fertilization improves microbial functional potentials for stress tolerance in arsenic–enriched rice cropping systems. J. Hazard. Mater..

[60] Das S., Hwang H.Y., Song H.J., Cho S.R., Van Nostrand J.D., Kim P.J. (2021). Soil microbial response to silicate fertilization reduces bioavailable arsenic in contaminated paddies. Soil Biol. Biochem..

[61] FAO Codex Alimentarius Commission-Geneva 14–18 July 2014. http://www.fao.org/news/story/en/item/238558/icode/.

[62] Bhattacharya S., Gupta K., Debnath S., Ghosh U.C., Chattopadhyay D., Mukhopadhyay A. (2012). Arsenic bioaccumulation in rice and edible plants and subsequent transmission through food chain in Bengal basin: A review of the perspectives for environmental health. Toxicol. Environ. Chem..

[63] Signes A., Mitra K., Burlo F., Carbonell-Barrachina A.A. (2008). Contribution of water and cooked rice to an estimation of the dietary intake of inorganic arsenic in a rural village of West Bengal, India. Food Addit. Contam..

[64] Golui D., Mazumder D.G., Sanyal S.K., Datta S.P., Ray P., Patra P.K., Sarkar S., Bhattacharya K. (2017). Safe limit of arsenic in soil in relation to dietary exposure of arsenicosis patients from Malda district, West Bengal—A case study. Ecotoxicol. Environ. Saf..

