# Supplementary material for: Co-Application of Silicate and Low-Arsenic-Accumulating Rice Cultivars Efficiently Reduces Human Exposure to Arsenic—A Case Study from West Bengal, India
Source: Toxics. 2023 Jan 9;11(1):64. doi: 10.3390/toxics11010064 (PMC9865337; doi:10.3390/toxics11010064)
Supplement: Supplementary file 1 [file toxics-11-00064-s001.zip › toxics-2055825-supplementary.pdf]

toxics

Co-Application of Silicate and Low-Arsenic-Accumulating Rice Cultivars Efficiently Reduces Human Exposure to Arsenic—A Case Study from West Bengal, India

Table S1. Operating conditions for ICP-MS (Model- Perkin Elmer, NexIon 300).

Component/Parameter	Type/Value/Mode
Nebulizer	PFA ST
Spray chamber	Peltier-cooled baffled quartz cyclonic
Triple cone interface material	Nickel
Plasma gas flow	16.0 L/min
Auxiliary gas flow	1.2 L/min
Nebulizer gas flow	0.98 L/min
Sample uptake rate	270 µL/min
RF power	1600 W
Analytes	Al, As, Cd, Cr, Cu, Pb, Mn, Ni, Se, Ag, Tl, V and Zn
Internal standards	Sc, In, Ge (added on line)
Modes of operation	1. Standard<br>2. Collision/KED (He gas)<br>3. Reaction (NH_3 gas)
Replicates per sample	3
Measurement time (3 replicates)	1 min 30 sec
Analysis time (sample to sample)	2 min 30 sec

Table S2. Carcinogenic risk in adult and children through consumption of dietary intake of rice grain.

Variety	Treatment	CR for adult	CR for children
IR-36	S0	0.002534	0.002628
	S1	0.002346	0.002433
	S2	0.002253	0.002336
Khitish	S0	0.001948	0.002020
	S1	0.001713	0.001776
	S2	0.001197	0.001241
Satabdi	S0	0.002839	0.002944
	S1	0.002464	0.002555
	S2	0.002229	0.002312
Badshabhog	S0	0.000774	0.000803
	S1	0.000188	0.000195
	S2	0.000188	0.000195
Mean		0.001723	0.001786
Max		0.002839	0.002944
Min		0.000188	0.000195
Std Dev		0.000881	0.000914

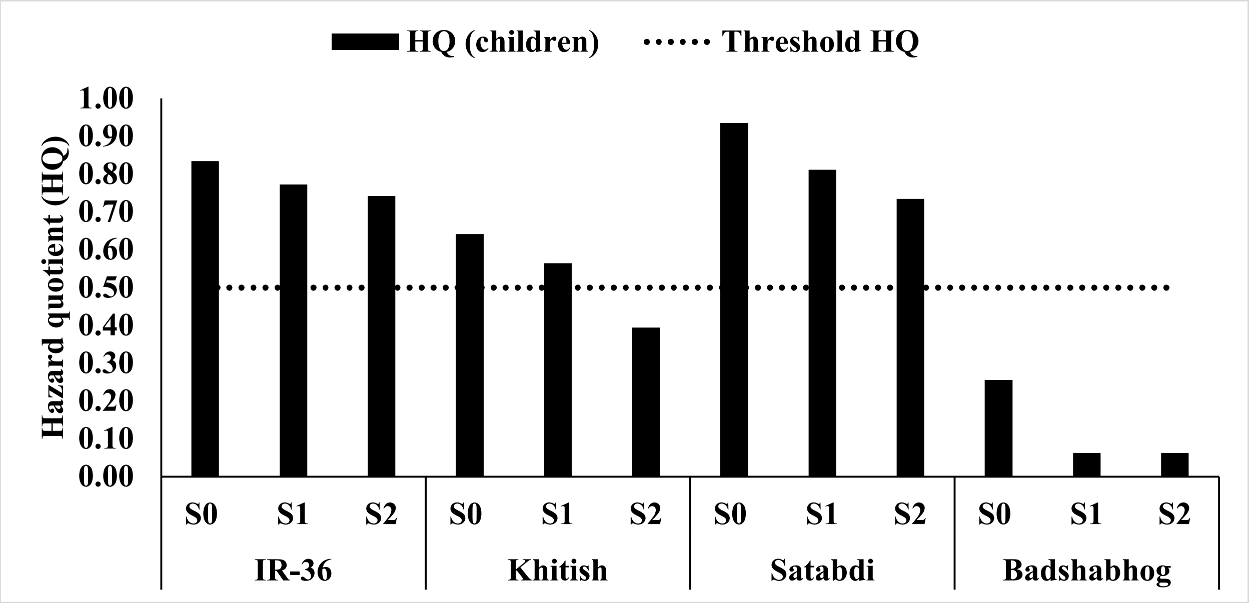

Figure S1. Hazard quotient values for arsenic in different rice cultivars under silicate treatments (Sodium metasilicate (Na_2SiO_3) was added to the soils in pot at the rates of 0 (S0), 250 (S1) and 500 (S2) mg kg^{-1} soil).
